# Spring diet and energy intake of tundra swan (*Cygnus columbianus*) at the Yellow River National Wetland in Baotou, China

**DOI:** 10.7717/peerj.13113

**Published:** 2022-03-15

**Authors:** Li Liu, Chao Du, Yan Sun, Wenjing Li, Jiyun Zhang, Litong Cao, Li Gao

**Affiliations:** Faculty of Biological Science and Technology, Baotou Teachers’ College, Baotou, Inner Mongolia, China

**Keywords:** Tundra swan, Yellow River National Wetland, Diet analysis, Energy supply

## Abstract

The Yellow River National Wetland in Baotou, China is an important resting and energy replenishment place for many migratory birds, such as tundra swan (*Cygnus columbianus*). The energy supply of food available at stopover sites plays an important role in the life cycle of migratory birds. In order to understand diet composition and energy supply of tundra swans for further protection of them, in this study, fecal of tundra swans (*C. columbianus*) were collected and fecal microhistological analysis was conducted to analyze the feeding habits and the energy supply. Results showed that: (1) tundra swans (*C. columbianus*) mainly fed on twelve species of plants from five families, including corn (*Zea mays*), quinoa (*Chenopodium album*) and rice (*Oryza sativa*), this is related to local crops and abundant plants. (2) The energy provided by crops to tundra swans (*C. columbianus*) was significantly higher than other abundant plants in wetlands (*P* < 0.05), corn and rice were the most consumed food, and other abundant wetland plants play complementary roles. (3) The daily energy intake of tundra swans (*C. columbianus*) was much higher than their daily energy consumption, the daily net energy intake of tundra swans (*C. columbianus*) was 855.51 ± 182.88 kJ (mean ± standard deviations). This suggested that the wetland provides energy for continue migrating to the tundra swan (*C. columbianus*). For further protection of tundra swans (*C. columbianus*) and other migratory birds, the Baotou Yellow River National Wetland environment and the surrounding farmland habitat should be protected.

## Introduction

During the migration of birds, they need to stop along the migration route to supply with food and accumulate energy to prepare for the next stage of flight. Therefore, the stopover sites are the hubs connecting the breeding and non-breeding sites of birds, and are of great significance for the migration of birds (Cao, Meng & Zhao, 2021). In addition, birds may face unfavorable environmental conditions such as severe cold and lack of food in the early stage when reaching the breeding grounds, so the energy reserved in the stopover areas also play an important role in the successfully reproduction of birds (Hua, Piersma & Ma, 2013; Wang et al., 2019). Food supplies at stopover sites sometimes influencing the subsequent survival and reproductive success of individuals, which can in turn affect subsequent breeding numbers (Newton, 2006). At these sites individuals must acquire sufficient body reserves of energy and nutrients to allow successful completion of the migration to the breeding grounds and subsequent reproduction. Therefore, stopovers play an important role in the complete life cycle of migratory birds (Stillman et al., 2015).

Food is the energy source of migratory birds, it is a vital factor influencing the number and distribution of bird populations (Chen et al., 2019). At the same time, waterbirds have been shown to track environmental variations, the abundance and distribution of waterbirds can reflect the structure and functions of wetlands, making them important bio-indicators for wetland health (Amat & Green, 2010; Yang et al., 2016). Wetlands possessing abundant vegetation and diverse animal and plant species, play a pivotal role in global biodiversity conservation. Yet in recent years wetlands are one of the most threatened ecosystems. A total of 35% of wetlands have been lost globally since 1970, wetland degradation will bring crisis to the survival of wild animals (Xu et al., 2019). At present, the loss of wetlands in eastern China due to urban expansion has threatened the migration of birds in East Asia (Murray et al., 2014; Mao et al., 2018). Due to the degradation and loss of natural wetlands, as well as the lack of food resources, a large number of waterbirds migrate to the farmland with rich food resources, making farmland an important foraging ground for waterbirds (Fox & Abraham, 2017). Since the mid-twentieth century, agricultural land has supported increasingly large numbers of avian herbivores (Newton, 2017). High-energy crops can provide important food resources for herbivores, especially during winter when the availability of natural vegetation is low (Clausen et al., 2018). Following their arrival on the wintering grounds, swans need to regain energy lost during earlier movements, and also to gain and maintain adequate energy reserves to allow them to survive winter and initiate subsequent migratory flights to their breeding grounds and reproduce successfully (Bêty, Gauthier & Giroux, 2003; Drent, Fox & Stahl, 2006). Such energy demands are particularly acute for long-distance migratory species such as Bewick’s swan (*Cygnus columbianus bewickii*) and whooper swans (*Cygnus cygnus*) (Wood et al., 2021). After waterfowl enter into the farmland, it brings new problems to the bird protection, such as the risk of interference by human activities, pesticide poisoning, predation and so on (Yu et al., 2017a). On the other hand, there is also a serious conflict between migratory bird protection and farmers due to the damage is alleged to growing grass, cereals and high value cash crops (Owen, 1990). In addition, because of the cultivated land itself is not directly protected, agricultural production is affected by government policies, economy and climate, it may change to the planting of crops that are not suitable for swans during a very short time, which brings more difficult challenges to its protection (Beekman et al., 2019; Fang et al., 2020). Populations of swans can show long-term declines in response to demographic and environmental factors (Beekman et al., 2019), and so it is critical that waterfowl and their habitats receive adequate protection (Li et al., 2020). Clarifying the food composition and energy supply of waterfowl is the key to formulate effective protection measures and is very important to maintain the population.

The Yellow River National Wetland in Baotou, China, is situated at an important intersection between East Asian/Australian and Central Asian flyways, abundant wetland resources provide sufficient food and suitable habitat for migratory birds (Li et al., 2017). During the migration season, tens of thousands of migratory birds, represented by swans, stop here to replenish energy (Liu et al., 2020). The tundra swans (*Cygnus columbianus*) arrived at the wetland to replenish energy in early February every year. The migration peaks reached in mid-March and gradually migrates away from the wetland in early April. Worldwide studies on the tundra swans mainly focused on the wintering habitat selection (Yu et al., 2019), behavioral ecology (Wood et al., 2019), migration strategy (Wang et al., 2019; Fang et al., 2020), etc., there are few studies on the energy supply of important resting places, which is not conducive to the conservation of the species and the maintenance of biodiversity.

The energy of migratory birds mainly comes from the fat stored in their bodies, and food resources are an important limiting factor of energy accumulation. Therefore, the energy required for migratory species is mainly determined by the quality of the resting place (Wang et al., 2019). This study aimed to clarify the food composition and energy supply of tundra swans (*C. columbianus*), which is a representative spring migration bird species, and reveal the environmental quality of the wetland. The results would provide an important scientific basis for the protection of this species, and would play a positive role in wetland ecological environment quality assessment and biodiversity maintenance.

## Materials and Methods

### Study area

Baotou Yellow River National Wetland (109°25′51″–111°1′36″E, 40°14′39″–40°33′20″N) is located to the south of Baotou, Inner Mongolia, China, and it lies on the north bank of the Yellow River, with a total length of about 220 km and a total area of 293.39 km^2^.

The wetland belongs to the arid to semi-arid temperate continental monsoon climate, its annual average temperature was 8.5 °C, the average annual precipitation was 240–400 mm, and the annual evaporation was 2,100–2,700 mm. The lowest temperature was about −34.4 °C reached in January, while the highest temperature was about 38.4 °C reached in July (Liu et al., 2020). Water level fluctuates greatly in this wetland. The freezing period is from January to February. River opening period is in March and has the highest water level in this period. Dry season was in summer. Due to the fluctuation, the wetland area expands during the ice flood season, and the floodplain formed by the large area of farmland surrounding provides sufficient food and suitable habitat for waterbirds such as tundra swans (*C. columbianus*) (Li et al., 2017). At the same time, large areas of emergent reed (*Phragmites australis*) grow around the wetlands, which are more concealed, providing suitable habitats for waterbirds.

The wetland is an important stopover site for migratory birds, there were 228 species of waterfowl in total (Yu et al., 2017b), of which 36 species were listed in the International Redbook. The dominant plants are reed (*P. australis*), suaeda (*Suaeda glauca*) and quinoa (*Chenopodium album*) (Liu et al., 2014). The main crops planted around the wetland are corn (*Zea mays*), wheat (*Triticum aestivum*) and rice (*Oryza sativa*) (Liu et al., 2020).

### Ethics Statement

The current study was performed in accordance with the recommendations on animal care and ethics of China. Noninvasive techniques were used to collect the fecal samples. The Animal Ethics and Welfare committee (AEWC) of Baotou Teachers College approved the implementation of the project. The management authority of Baotou Yellow River National Wetland approved the collection of tundra swans (*C. columbianus*) fecal samples.

### Sample collection

A total of 100 fecal samples were collected at four sites in the wetland from late February to mid-March, 2020. Sampling sites with large flocks of tundra swans (*C. columbianus*) (*i.e*., more than 200 individuals) were chosen based on the previous studies and the latest survey of the waterbird. Generally, tundra swans (*C. columbianus*) leave feces at sleeping sites. The sleeping position was investigated on the first night. The resting position was confirmed again the next morning, and fresh fecal samples were collected after the tundra swans (*C. columbianus*) had left. The surface detritus of the collected fecal samples were removed and stored in paper bags. Disposable gloves were changed for each sample to avoid cross-contamination. Each sample was collected from sleeping positions separated by more than 2 m to avoid repeated sampling and ensure that each samples was from different individuals. All samples were collected and stored in paper bags, transported to laboratory and stored at −20 °C until analysis. On the day the fecal samples were collected, potential food plants were collected around the tundra swans’ foraging grounds, marked, and brought back to the laboratory for identification.

### Diet composition

We performed microhistologic examination of fecal samples using the method described by Owen (1975). Epidermal fragments of plants are not easily digested after passing through the digestive tracts of herbivorous birds, for example, the digestive efficiency of swans feeding on grasses was only 21% (Wood et al., 2013); therefore, feeding habits can be analyzed based on excrement. The samples were dried at 50 °C in a drying oven (BPG-9140A, China) for 48 h, then 20 samples from each site were randomly chosen and grounded to powder, eluted with water, mixed thoroughly, and examined under a microscope (BA410E; Motic, China).

The collected plant samples were divided into seeds, stems, and leaves, crushed to a debris with a mortar, and dried using the same drying method employed for the fecal samples. Then, the fragments in the fecal samples were compared with the plant specimens collected from the foraging sites and examined under a light microscope at 40× magnification for species identification and 10× magnification for quantification statistics.

Diet composition was quantified by measuring the relative frequency and density of the fragments (Johnson, Wofford & Pearson, 1983), and i represents the proportion of plant species in the food, which can be expressed as:

(1) $$F = 1 - {e^{ - d}}$$

where *F* is the relative frequency, *e* is the natural logarithm, *d* is the mean fragment density, which can be determined by the number of fragments (*n*) and the number of microscope fields examined (*k*) as follows:

(2) $$d = {n \over k}$$

Fragments from m different plant species were randomly distributed in the microscope fields, and the average density of each plant (average number of fragments per field) was not relevant to other species. The relative particle densities (*r_i_*), which were estimated by the relative dry weights of each plant in the diet sample, was calculated using the following formula:

(3) $${r_i} = {{{d_i}} \over {\sum\limits_{i = 1}^m {{d_i}} }}$$

where i = l,…, m, and *d*_*i*_ are the average densities for each species.

### Estimating energy budgets

We calculated the daily food intake based on the body weight of tundra swans (*C. columbianus*) (Nagy, 1987). The energy intake of food and the energy excreted from the feces was estimated according to the weight percentage (relative density) of each plant in the diet, the energy (*Q*_*i*_) of each plant, and the indigestible endogenous marker acid detergent fiber (ADF) of the plants. The difference between energy intake and excretion was the daily energy consumption of tundra swans (*C. columbianus*). The daily energy consumption of tundra swans was estimated according to the observation of the daily activity rule and individual body temperature. The difference between energy intake and energy consumption is the daily net energy intake of tundra swans (*C. columbianus*).

#### Estimating daily metabolizable energy intake

To estimate daily metabolizable energy intake (*MEI*), the fecal digestion efficiency and energy content of food plants and droppings need to be estimated. Digestion efficiency was estimated by calculating the relative concentration of the indigestible marker acid detergent fiber (ADF) in the food and feces (Durant, 2003).

The total energy contents of the droppings and the plants were mesured by aParr® 6100 calorimeter (PARR, Moline, IL, USA), and the metabolizable energy intake was estimated.

The daily feed intake of tundra swans (*C. columbianus*) is calculated as follows:

(4) $${I_{df}} = 0.648B{W^{0.651}}$$

where *I*_*df*_ is the food consumption rate per day (dry weight, g d^−1^) (food consumption rates were estimated using allometric regression models) (Nagy, 1987), and *BW* refers to body weight (g). *BW* of adult tundra swans (*C. columbianus*) was 6,000 g (Nolet & Klaassen, 2005) Multiplying the value obtained by the proportion of food and energy, the daily energy intake (*DEI*) of tundra swans (*C. columbianus*) was calculated as follows:

(5) $$DEI = \sum\limits_{i = 1}^m {{I_{df}}} \cdot {r_i} \cdot {Q_i}$$

where, *Q*_*i*_ is the energy of the plant per unit mass *i*.

The energy excreted from feces was estimated by multiplying the daily total defecation mass (*m*_*T*_) by the caloric values of dropping per unit mass (*Q*_*d*_) (kJ g^−1^ dry weight). The total daily defecation mass was calculated according to the mean dropping interval (*t*_*d*_), average dropping mass (*m*_*d*_), and total time spent active (*t*_*A*_). *m*_*T*_ was calculated by the method described by Wang et al. (2013a):

(6) $${m_T} = {{{t_A}} \over {{t_d}}} \cdot {m_d}$$

We directly observed the fecal interval of cygnets by recording the time interval between two randomly selected consecutive faeces. Get at least 30 time intervals per week and average them as *t*_*d*_.

The metabolizable energy intake (*MEI*) was calculated as follows:

(7) $$MEI = DEI - {Q_d} \cdot {m_T}$$

#### Estimating daily energy expenditure

Daily energy expenditure was gauged by integrating the different behaviors’ energy cost based on the basal metabolic rate (*BMR*, kJ h^−1^), and calculated according to the methods described by Reynolds & Lee (1996) as follows:

(8) $$BMR = 0.176 \times B{W^{0.635}}$$

The weight of the tundra swans (*C. columbianus*) in this study refers to the weight (6,000 g) described by Nolet & Klaassen (2005). The energy expenditure for flying, feeding, alert, resting (sleeping, standing, sitting, and floating combined) and other activities (swimming, drinking and so on) were 23.46 × BMR, 1.52× BMR, 1.61 × BMR, 1.26 × BMR and 3.61 × BMR, respectively (Wood et al., 2021). These values were almost the same as pink-footed goose (*Anser brachyrhynchus*), Greater White-fronted Geese (*Anser albifrons*) studied by other methods (Madsen, 1985;Mooij, 1992).

Low temperatures increase energy expenditure. Tundra swans (*C. columbianus*) must increase their ability of heat production to maintain body temperature below the ‘lower critical temperature’ (*LCT*, °C). The *LCT* was calculated using a formula reported by Calder & King (1974):

(9) $$LCT = {T_0} - 4.73 \times {W^{0.274}}$$

In the above formula, body temperature (T_0_) was assumed to be 40 °C. Daily heat loss below the *LCT* (*H*_*LCT*_, kJ d^−1^) was calculated by the method described by Lefebvre & Raveling (1967):

(10) $${H_{LCT}} = a \times ({T_b} - LCT) \times {H_b}$$

The coefficient ‘a’ (2.18) was estimated based on data provided by Lefebvre & Raveling (1967) using a quadratic regression of the heat loss coefficient against body mass. *T*_*b*_ and *H*_*b*_ were respectively the average temperature and hours below the *LCT*. We used the daily maximum and minimum temperatures of Baotou, approximately 30 km away from the study area.

### Analysis and statistics

In order to understand the differences in energy provided by different plants in the tundra swan’s diet, the proportional food provides energy ratio was arcsine square root transformed and analyzed using principal components analysis (PCA) using SPSS v. 20.0. Each sample collection value was then plotted according to its loading on the first two axes of the PCA. The difference in energy supply to tundra swans (*C. columbianus*) between crops and the most abundant wetland plants was analyzed using a non-parametric Kruskal-Wallis test (*P* < 0.05) (SPSS). The differences in feeding frequency among different plants were analyzed using a one-way ANOVA.

## Results

### Food composition

The results of the diet analysis showed that (Table 1) the tundra swan (*C. columbianus*) fed mainly on twelve species of plants from five families at the Yellow River National Wetland in Baotou. Corn (*Z. mays*) (31.38%), quinoa (*C. Album*) (18.21%), rice (*O. sativa*) (16.68%), millet (*Panicum miliaceum*) (13.49%) were the most abundant component of the diet. Followed by kochia (*Kochia scopavia*) (5.47%), polygonum (*Polygonum hydropiper*) (4.62%) and reed (*P. australis*) (3.77%) (% refers to the relative particle densities (*ri*), which were estimated by the relative dry weights of each plant in the food composition). Analysis by one-way ANOVA of the feeding frequency of the five families showed that the Graminaeae was significantly higher than the other families (*P* = 0.00, *d_f_* = 79). Further analysis showed that the feeding frequencies of crops was significantly higher than that of the dominant plants in the wetland (*P* = 0.00, *d*_*f*_ = 79).

**Table 1:** Food composition of tundra swan.

Family	Plant species	Feeding part	Relative frequency (%)	Relative density (%)
Graminaeae	*Zea mays*	Seed	25.84	31.38
		Leaf	1.48	
	*Oryza sativa*	Seed	14.74	16.68
		Leaf	0.74	
	*Panicum miliaceum*	Seed	10.05	13.49
		Leaf	1.97	
		Stem	0.92	
	*Phragmites australis*	Leaf	3.70	3.77
Chenopodiaceae	*Chenopodium album*	Seed	16.65	18.21
	*Kochia scopavia*	Seed	5.33	5.47
	*Chenopodium urbicum*	Seed	3.36	3.41
	*Suaeda glauca*	Stem	0.24	0.24
Polygonaceae	*Polygonum hydropiper*	Leaf	4.51	4.62
Compositae	*Ixeris denticulata*	Leaf	0.78	0.78
	*Xanthium sibiricum*	Leaf	0.49	0.49
Labiatae	*Clinopodium chinense*	Seed	0.39	0.65
		Stem	0.26	
	Others		0.77	0.77

### Analysis of plant energy contribution

To clarify the energy supply of various plants to tundra swans (*C. columbianus*), PCA analysis was carried out on the energy values of the twelve most abundant plants in droppings (Table 2). The first two PCA axes accounted for 45.36% (29.62 and 15.74%, respectively) of the energy contribution. The first axis was characterized by the high positive loadings of corn (*Z. mays*), and the high negative loadings of quinoa (*C. album*) and millet (*P. miliaceum*). The second axis was characterized by the high positive loadings of rice (*O. sativa*), and the high negative loadings of polygonum (*P. hydropiper*) and suaeda (*S. glauca*).

**Table 2:** The major plant species identified in tundra swan fecal matter and their corresponding energy loadings.

Plant species	Eigenvector axis 1	Eigenvector axis 2
*Zea mays*	**0.810**	−0.450
*Panicum miliaceum*	**−0.694**	−0.219
*Oryza sativa*	0.353	**0.710**
*Phragmites australis*	0.542	0.426
*Chenopodium album*	**−0.776**	−0.009
*Kochia scopavia*	0.611	0.186
*Chenopodium urbicum*	−0.663	0.114
*Polygonum hydropiper*	−0.356	**−0.569**
*Ixeris denticulata*	−0.074	−0.397
*Xanthium sibiricum*	0.122	−0.542
*Clinopodium chinense*	0.458	0.270
*Suaeda glauca*	−0.068	**0.529**

**Note:**

The values in the table represent the eigenvectors calculated for each species. Values in bold font indicate the three plant species that contributed the most to each axis.

**Figure 1:**
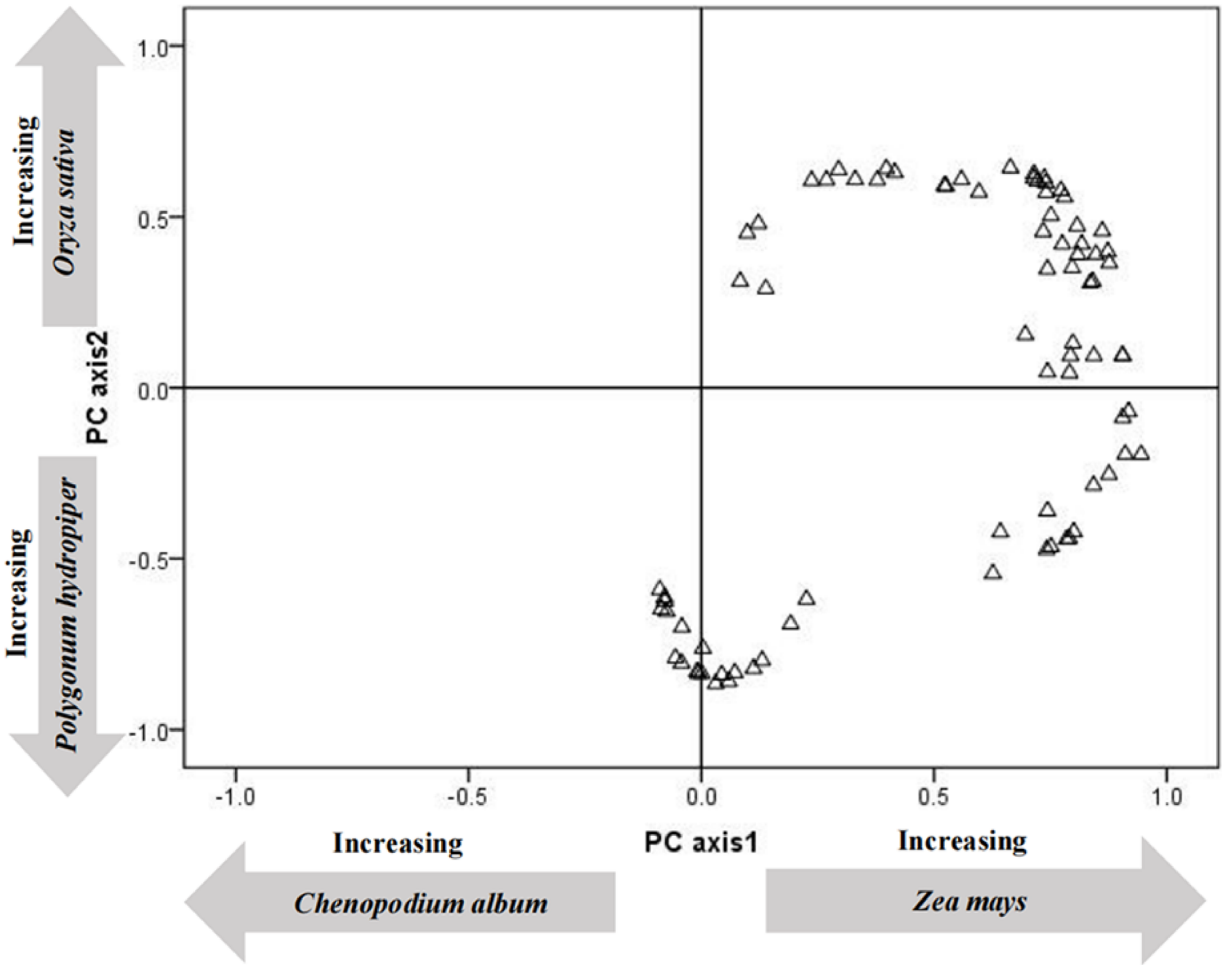
Principal components analysis ordination of the content of collections of fecal.

The load distribution map of all the energy contributions of each plant species (Fig. 1) was drawn using the first two principal components as the coordinate axes. Results showed that the main energy sources for tundra swans (*C. columbianus*) at this point in their migration were crops and abundant plants. To clarify the difference in energy provided by wetland plants and crops with the same weights, a non-parametric test was conducted on all plant species in terms of food composition. Results showed that the energy provided by crops was significantly higher than that derived from the most abundant wetland plants (*Z* = −2.391, *P* = 0.017, *d*_*f*_ = 11).

### Daily metabolizable energy intake and consumption

Results showed that corn (*Z. mays*) seeds, quinoa (*C. album*) seeds and rice (*O. sativa*) seeds provided the highest daily average intake of food energy for the tundra swan (*C. columbianus*), the food energy they provided were 893.83 ± 15.62 kJ d^−1^, 435.99 ± 2.73kJ d^−1^ and 413.14 ± 0.06 kJ d^−1^, respectively (Table 3). The daily metabolizable energy intake (*MEI*) of tundra swan (*C. columbianus*) was calculated to be 2,519.31 ± 11.59 kJ. The excretion energy of feces is 518.26 ± 0.77 kJ d^−1^, so the daily metabolizable energy intake of the tundra swan (*C. columbianus*) is 2,001.05 ± 10.82 kJ d^−1^, The energy supports a variety of behavioral activities, including flying, feeding, alerting, and resting. The energy consumption was estimated to be 1,145.54 ± 183.70 kJ. The behavior of tundra swan’s daytime energy expenditure is 289.30 ± 81.94 kJ d^−1^ resting, 177.98 ± 74.84kJ d^−1^ for feeding, and 380.90 ± 26.91kJ d^−1^ for flying. The difference between daily metabolizable energy intake and daily energy consumption is the daily net energy intake of the tundra swan (*C. columbianus*), and we found this to be 855.51 ± 182.88 kJ (Note: Data indicate mean ± standard deviations).

**Table 3:** The daily energy intake of tundra swan to different plants.

Plant species	Feeding part	Total energy (kJ g^−1^)	ADF (%)	Daily energy intake (kJ d^−1^)
*Zea mays*	Seed	16.68	3.98	893.83 ± 15.62
	Leaf	17.22	35.18	31.09 ± 0.19
*Oryza sativa*	Seed	16.43	15.51	413.14 ± 0.06
	Leaf	15.10	43.50	11.83 ± 0.09
*Panicum miliaceum*	Seed	17.37	25.23	256.91 ± 0.48
	Leaf	16.08	38.87	36.42 ± 0.16
	Stem	16.32	43.18	16.00 ± 0.07
*Phragmites australis*	Leaf	18.84	35.45	85.59 ± 0.71
*Chenopodium album*	Seed	17.62	27.23	435.99 ± 2.73
*Kochia scopavia*	Seed	19.89	27.71	4.36 ± 0.25
*Chenopodium urbicum*	Seed	18.04	25.99	146.92 ± 0.12
*Suaeda glauca*	Stem	14.73	37.19	85.09 ± 0.04
*Polygonum hydropiper*	Leaf	16.19	54.60	63.41 ± 0.38
*Ixeris denticulata*	Leaf	17.99	36.08	16.75 ± 0.22
*Xanthium sibiricum*	Leaf	15.94	26.76	10.75 ± 0.12
*Clinopodium chinense*	Seed	19.15	43.24	7.98 ± 0.12
	Stem	17.24	61.39	3.26 ± 0.07

## Discussion

The previous studies showed that the types of plants that the tundra swan (*C. columbianus*) eats varied from different time and region, showing diversity characteristics. Wood et al. (2019) found that the tundra swan of the north-west European population mainly ate beets (*Beta vulgaris*) and potatoes (*Solanum tuberrosum*) during the overwintering period, while crop wheat (*T. aestivum*) at the end of the overwintering period. Yu et al. (2019) found that tundra swans (*C. columbianus*) mainly fed on gorgon (*Euryale ferox Salisb*) and rice (*O. sativa*) in the middle and lower Yangtze River, China. Cong et al. (2011) found that tundra swans (*C. columbianus*) in the Yangtze River floodplain mainly fed on bitter grass (*Vallisneria natans*). In the present study, we analyzed the feeding habits of tundra swans (*C. columbianus*) in Baotou Yellow River, results showed that the tundra swans (*C. columbianus*) mainly fed on corn (*Z. mays*), quinoa (*C. album*), millet (*P. miliaceum*) *and* rice (*O. sativa*). The food composition was associated with the local crops and the abundant wetland plants. The selection of food diversity reflects the adaptation of waterfowl to the environment. In addition, understanding how avian herbivores respond to changes in the availability of food resources, which may be caused by reduced availability of key crop types or increased competition with other herbivores, is essential for the conservation of these species (Wood et al., 2021). When investigating the food resources of the Yellow River wetlands, we found that large areas of the Yellow River beaches were reclaimed and planted with crops such as corn (*Z. mays*) and rice (*O. sativa*). During the icy flood season, a large number of crops were submerged by the rising water of the Yellow River, and they were sealed by ice throughout the winter. After the thawing of the river, a large number of crops were exposed, providing food resources for the tundra swans (*C. columbianus*) migrating in the spring (Liu et al., 2014). So we thought that abundant food resources were an important reason for the migratory birds to stop here to replenish energy.

According to the results of the current study, migratory birds can gain more energy from shifting their habitat from wetlands to farmland, this was also consist with most of the previous studies conducted in other areas and other bird species. Fox & Abraham (2017) found that farmland provides far more food resources for geese than the traditional natural wetlands, especially corn (*Z. mays*) seeds contained high level of protein and fat, which can provide a lot of energy for the geese. Yu et al. (2017a) pointed out that the shift from natural habitat to farmland for geese can greatly increase the rate of food intake, so that geese can attain higher physical conditions throughout the annual cycle and increase reproductive outputs, and the carrying capacity of non-breeding areas can also be expanded (Yu et al., 2017a). Satellite tracking results also confirmed that the main habitat of tundra swans (*C. columbianus*) in the spring migration season is the mudflats formed by the flooded farmland of the Yellow River (Huang et al., 2018). Although there are also some studies showing that some waterbirds are reluctant to develop farmland (Zhao et al., 2018). In the current study, it was found that among the food components, the crop corn (*Z. mays*), rice (*O. sativa*), and millet (*P. miliaceum*) were the most frequently ingested by tundra swan (*C. columbianus*) and accounted for more than 50% of all the food component. Energy supply analysis showed that the energy load factor contributed by crop was higher than that of other abundant wetland plants, indicating that crops played a leading role in the energy supply of tundra swans (*C. columbianus*) at this study site. Baotou Yellow River National Wetland is an important refuelling areas for tundra swans (*C. columbianus*). During the migration here, crops are mainly used. It may because that the native plants of the wetland are insufficient to provide energy supply for the migration of a large number of migratory birds, so they turn to farmland to feed on crops with higher energy and easy access. Therefore, in order to protect tundra swans (*C. columbianus*), it is important to protect the important refuelling areas, Baotou Yellow River National Wetland.

In addition, energy contribution analysis showed that abundant wetland plants (*Z. mays*, *O. sativa*), and millet (*P. miliaceum*) also provided an important energy supply for the tundra swan (*C. columbianus*), although their feeding frequency were relatively lower than crops, resulting to an overall limited energy source. We found that large areas of native wetlands in the study area were replaced by farmland, resulted in the usage of a large amount of herbicides, which degraded the function of the original wetland and reduced the number of dominant plants in the wetland (Xu et al., 2019), so the feeding frequency of these dominant plants reduced, this may also be the reason why the abundant wetland plants provide less energy than crops.

Migration of migratory birds is a process of rapid energy consumption. Birds need to store a large amount of fat to ensure their energy requirements during migratory fly, especially for long-distance migration Anseriformes. Wang et al. (2013b) discovered that the Lesser White-fronted geese (*Anser erythropus)* wintering in East Dongting Lake had the similar behavior with other wintering geese (Wang et al., 2013b). They accumulated energy in autumn, consumed energy in wint3er, and re-accumulated energy in spring to prepare for spring migration. Tinkler, Montgomery & EIwood (2009) analyzed the foraging ecology and energy of Canadian wintering brent geese (*Branta bernicla*), pointed out that energy stored at the end of the winter can power for migration (Tinkler, Montgomery & EIwood, 2009). Chen et al. (2019) analyzed that the Barnacle geese (*Branta leucopsis*) overwintering in Shengjin Lake was fed on the tubers of submerged plant *Vallisneria* buried in the ground. They pointed out that Anatidae species usually adopt energy maximization strategies. Barnacle geese (*B. leucopsis*) maximized daily energy intake by digging plant tubers of medium depth (11–20 cm) to store energy for migration. Our results showed that the daily energy intake of tundra swans (*C. columbianus*) is 2,519.31 ± 11.59 kJ, which is higher than the total energy expenditure of 1,145.54 ± 183.70 kJ. Similar to the previous results, there was also an accumulation of energy for tundra swans (*C. columbianus*) in the Yellow River National Wetland in Baotou, China, which can provide the power to continue the migration.

Baotou Yellow River National Wetland is surrounded by a large area of farmland, and pesticides (*i.e*., herbicides) are inevitably used in agricultural production activities. Excessive use of pesticides poses risks to reduce native plants in wetland and negative health impacts on human and migratory birds. To better protect the health of humans and migratory birds, biological control should be strengthened. In addition, due to the large-scale reclamation of the Yellow River for planting crops, the natural wetland area is reduced, the habitat of migratory birds is lost, and the influence of human activities is increased. In order to protect migratory birds, the original wetland area should be preserved as far as possible.

## Conclusions

We analyzed the diet composition and energy supply of tundra swans (*C. columbianus*) in Baotou Yellow River National Wetland, and found that tundra swans (*C. columbianus*) mainly fed on twelve species of plants from five families, including corn (*Z. mays*), quinoa (*C. album*) and rice (*O. sativa*), this is related to local crops and abundant plants. The energy provided by crops to tundra swans (*C. columbianus*) was significantly higher than other abundant plants in wetlands, corn (*Z. mays*) and rice (O, sativa) were the most consumed food, and other abundant wetland plants play complementary roles. The daily energy intake of tundra swans (*C. columbianus*) was much higher than their daily energy consumption, the daily net energy intake of tundra swans (*C. columbianus*) was 855.51 ± 182.88 kJ. This shows that Baotou Yellow River National Wetland is an important stopover sites for tundra swans (*C. columbianus*), which provides energy for their continued migration. In addition, after entering the farmland, the tundra swans (*C. columbianus*) faces the risks of human activity interference, pesticide poisoning, crop (food resources) change and so on. In order to protect the tundra swans (*C. columbianus*), the Baotou Yellow River National Wetland environment and the surrounding farmland habitat should be protected.

## Supplemental Information

10.7717/peerj.13113/supp-1 Supplemental Information 1 Raw data for food composition analysis.

## Additional Information and Declarations

### Field Study Permissions

The following information was supplied relating to field study approvals (*i.e*., approving body and any reference numbers): The current study was performed in accordance with the recommendations on animal care and ethics of China. Noninvasive techniques were used to collect the fecal samples. The Animal Ethics and Welfare committee (AEWC) of Baotou Teachers College approved the implementation of the project. The management authority of Baotou Yellow River National Wetland approved the collection of tundra swans (*C. columbianus*) fecal samples.

### Data Availability

The following information was supplied regarding data availability: The raw measurements are available in the Supplementary File.
